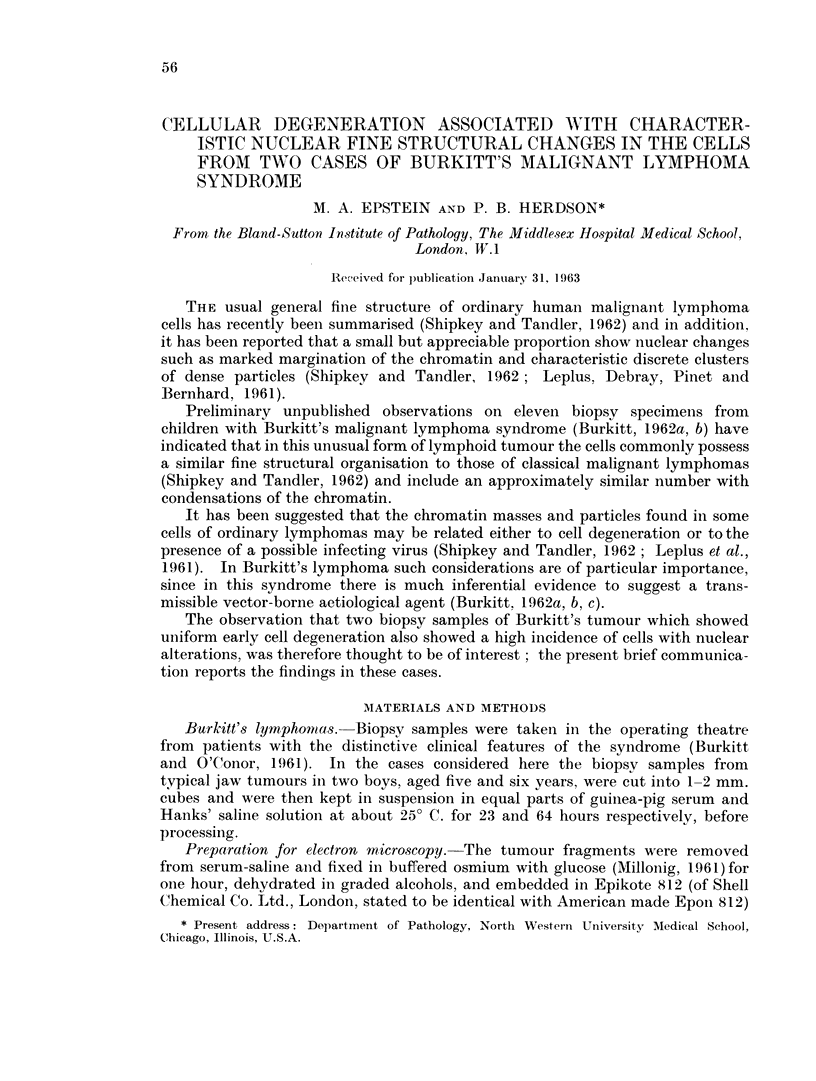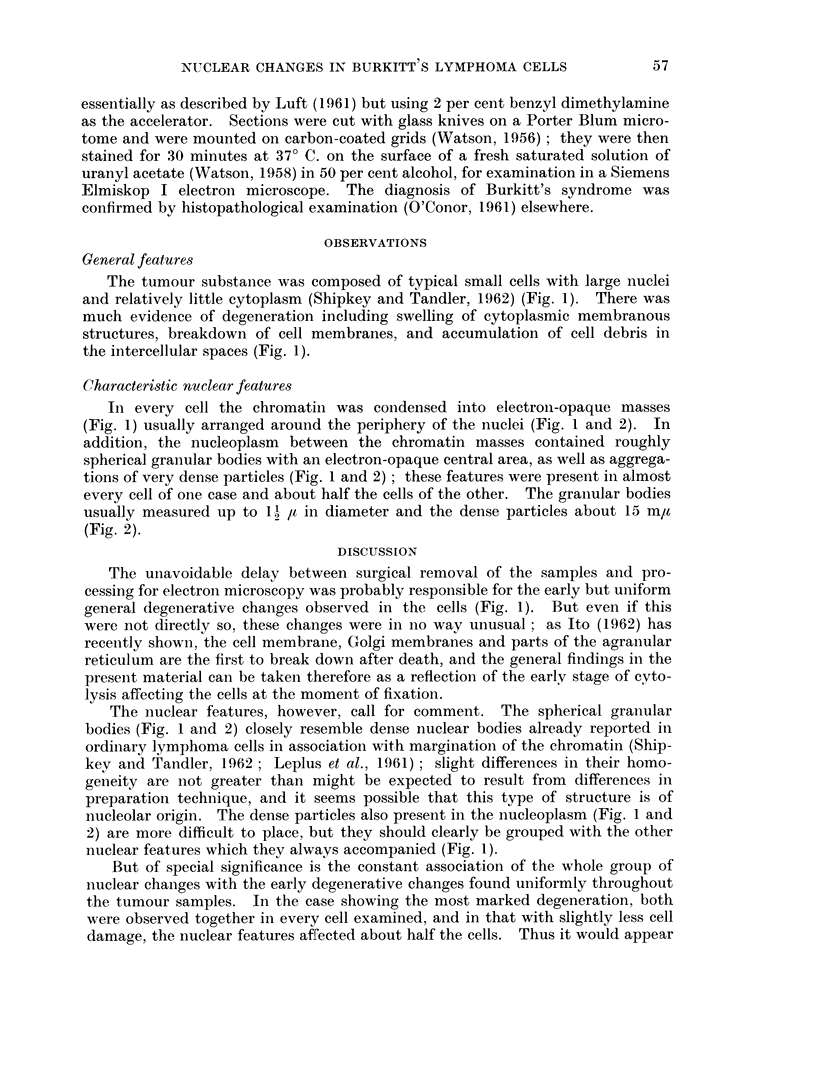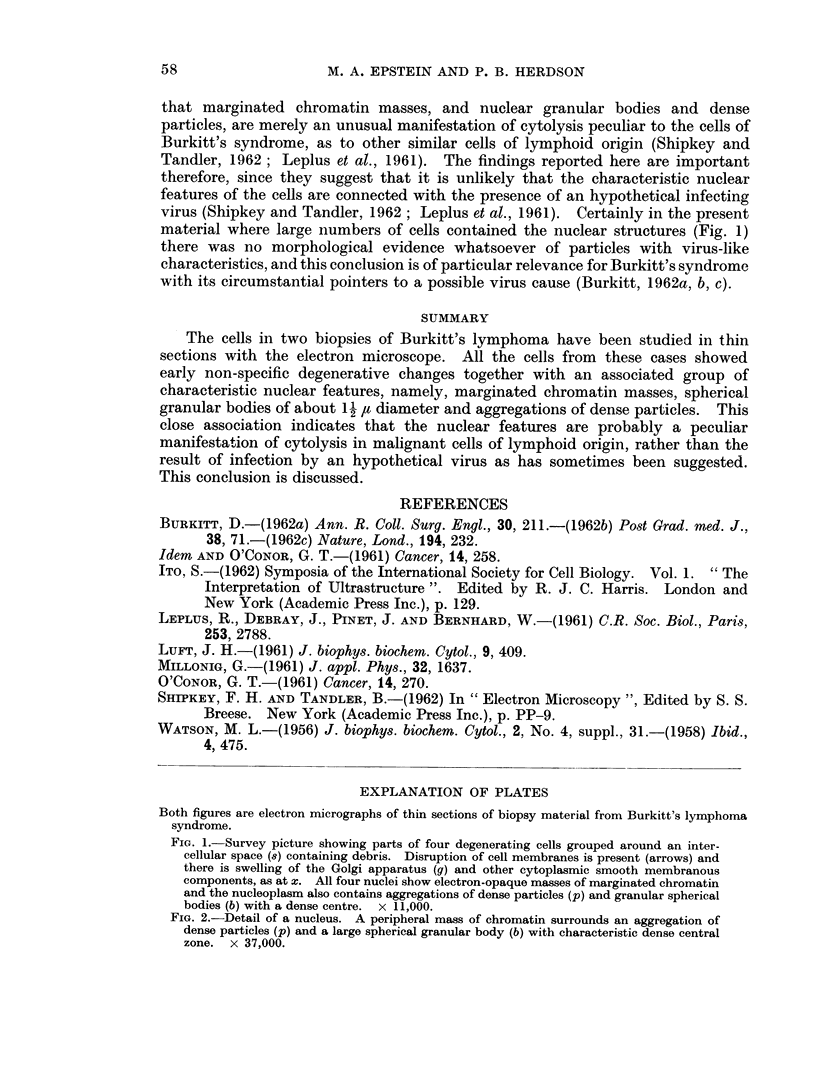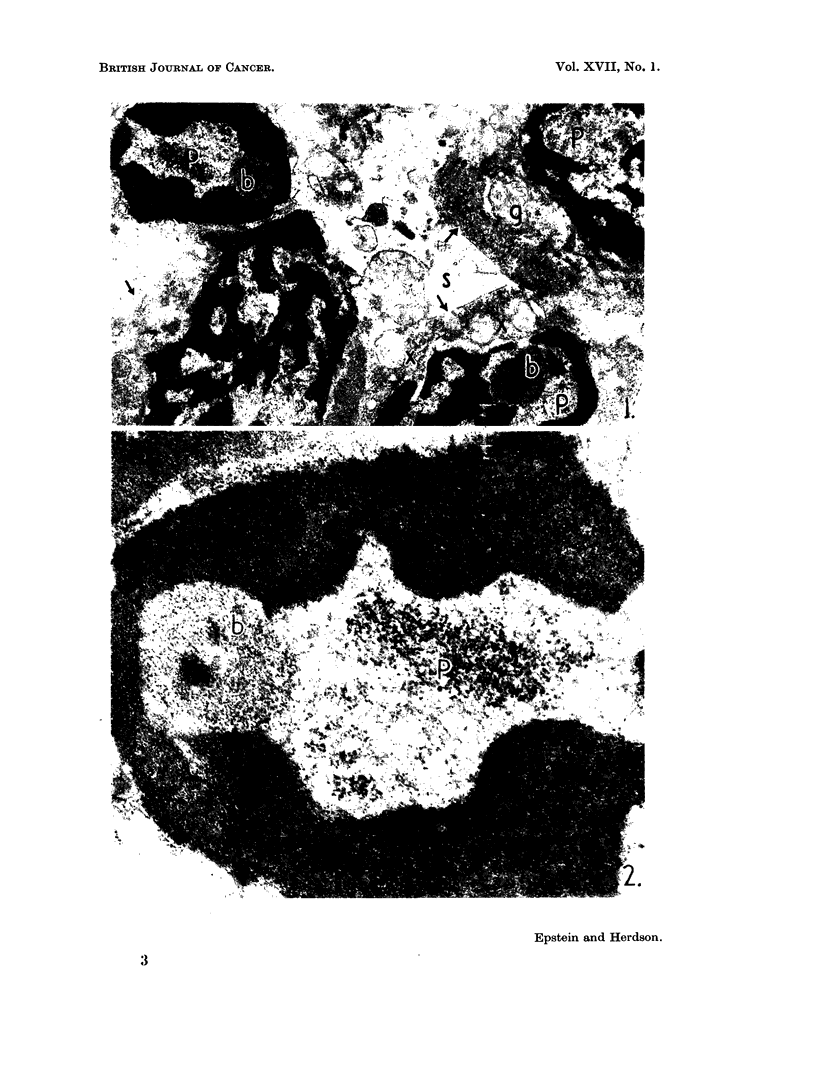# Cellular Degeneration Associated with Characteristic Nuclear Fine Structural Changes in the Cells from two cases of Burkitt's Malignant Lymphoma Syndrome

**DOI:** 10.1038/bjc.1963.8

**Published:** 1963-03

**Authors:** M. A. Epstein, P. B. Herdson

## Abstract

**Images:**


### CELLULAR DEGENERATION ASSOCIATED WITH CHARACTERISTIC NUCLEAR FINE STRUCTURAL CHANGES IN THE CELLS FROM TWO CASES OF BURKITT'S MALIGNANT LYMPHOMA SYNDROME

M. A. EPSTEIN AND P. B. HERDSON*

*From the Bland-Sutton Institute of Pathology, The Middlesex Hospital Medical School, London, W.1*

Received for publication January 31, 1963

THE usual general fine structure of ordinary human malignant lymphoma cells has recently been summarised (Shipkey and Tandler, 1962) and in addition, it has been reported that a small but appreciable proportion show nuclear changes such as marked margination of the chromatin and characteristic discrete clusters of dense particles (Shipkey and Tandler, 1962 ; Leplus, Debray, Pinet and Bernhard, 1961).

Preliminary unpublished observations on eleven biopsy specimens from children with Burkitt's malignant lymphoma syndrome (Burkitt, 1962*a*, *b*) have indicated that in this unusual form of lymphoid tumour the cells commonly possess a similar fine structural organisation to those of classical malignant lymphomas (Shipkey and Tandler, 1962) and include an approximately similar number with condensations of the chromatin.

It has been suggested that the chromatin masses and particles found in some cells of ordinary lymphomas may be related either to cell degeneration or to the presence of a possible infecting virus (Shipkey and Tandler, 1962 ; Leplus *et al.*, 1961). In Burkitt's lymphoma such considerations are of particular importance, since in this syndrome there is much inferential evidence to suggest a transmissible vector-borne aetiological agent (Burkitt, 1962*a*, *b*, *c*).

The observation that two biopsy samples of Burkitt's tumour which showed uniform early cell degeneration also showed a high incidence of cells with nuclear alterations, was therefore thought to be of interest ; the present brief communication reports the findings in these cases.

### MATERIALS AND METHODS

*Burkitt's lymphomas.*—Biopsy samples were taken in the operating theatre from patients with the distinctive clinical features of the syndrome (Burkitt and O'Conor, 1961). In the cases considered here the biopsy samples from typical jaw tumours in two boys, aged five and six years, were cut into 1–2 mm. cubes and were then kept in suspension in equal parts of guinea-pig serum and Hanks' saline solution at about 25° C. for 23 and 64 hours respectively, before processing.

*Preparation for electron microscopy.*—The tumour fragments were removed from serum-saline and fixed in buffered osmium with glucose (Millonig, 1961) for one hour, dehydrated in graded alcohols, and embedded in Epikote 812 (of Shell Chemical Co. Ltd., London, stated to be identical with American made Epon 812)

* Present address: Department of Pathology, North Western University Medical School, Chicago, Illinois, U.S.A.

essentially as described by Luft (1961) but using 2 per cent benzyl dimethylamine as the accelerator. Sections were cut with glass knives on a Porter Blum microtome and were mounted on carbon-coated grids (Watson, 1956) ; they were then stained for 30 minutes at 37° C. on the surface of a fresh saturated solution of uranyl acetate (Watson, 1958) in 50 per cent alcohol, for examination in a Siemens Elmiskop I electron microscope. The diagnosis of Burkitt's syndrome was confirmed by histopathological examination (O'Conor, 1961) elsewhere.

### OBSERVATIONS

### *General features*

The tumour substance was composed of typical small cells with large nuclei and relatively little cytoplasm (Shipkey and Tandler, 1962) (Fig. 1). There was much evidence of degeneration including swelling of cytoplasmic membranous structures, breakdown of cell membranes, and accumulation of cell debris in the intercellular spaces (Fig. 1).

### *Characteristic nuclear features*

In every cell the chromatin was condensed into electron-opaque masses (Fig. 1) usually arranged around the periphery of the nuclei (Fig. 1 and 2). In addition, the nucleoplasm between the chromatin masses contained roughly spherical granular bodies with an electron-opaque central area, as well as aggregations of very dense particles (Fig. 1 and 2) ; these features were present in almost every cell of one case and about half the cells of the other. The granular bodies usually measured up to $1\frac{1}{2}$ $\mu$ in diameter and the dense particles about 15 m$\mu$ (Fig. 2).

### DISCUSSION

The unavoidable delay between surgical removal of the samples and processing for electron microscopy was probably responsible for the early but uniform general degenerative changes observed in the cells (Fig. 1). But even if this were not directly so, these changes were in no way unusual ; as Ito (1962) has recently shown, the cell membrane, Golgi membranes and parts of the agranular reticulum are the first to break down after death, and the general findings in the present material can be taken therefore as a reflection of the early stage of cytolysis affecting the cells at the moment of fixation.

The nuclear features, however, call for comment. The spherical granular bodies (Fig. 1 and 2) closely resemble dense nuclear bodies already reported in ordinary lymphoma cells in association with margination of the chromatin (Shipkey and Tandler, 1962 ; Leplus *et al.*, 1961) ; slight differences in their homogeneity are not greater than might be expected to result from differences in preparation technique, and it seems possible that this type of structure is of nucleolar origin. The dense particles also present in the nucleoplasm (Fig. 1 and 2) are more difficult to place, but they should clearly be grouped with the other nuclear features which they always accompanied (Fig. 1).

But of special significance is the constant association of the whole group of nuclear changes with the early degenerative changes found uniformly throughout the tumour samples. In the case showing the most marked degeneration, both were observed together in every cell examined, and in that with slightly less cell damage, the nuclear features affected about half the cells. Thus it would appear that marginated chromatin masses, and nuclear granular bodies and dense particles, are merely an unusual manifestation of cytolysis peculiar to the cells of Burkitt's syndrome, as to other similar cells of lymphoid origin (Shipkey and Tandler, 1962 ; Leplus *et al.*, 1961). The findings reported here are important therefore, since they suggest that it is unlikely that the characteristic nuclear features of the cells are connected with the presence of an hypothetical infecting virus (Shipkey and Tandler, 1962 ; Leplus *et al.*, 1961). Certainly in the present material where large numbers of cells contained the nuclear structures (Fig. 1) there was no morphological evidence whatsoever of particles with virus-like characteristics, and this conclusion is of particular relevance for Burkitt's syndrome with its circumstantial pointers to a possible virus cause (Burkitt, 1962*a*, *b*, *c*).

### SUMMARY

The cells in two biopsies of Burkitt's lymphoma have been studied in thin sections with the electron microscope. All the cells from these cases showed early non-specific degenerative changes together with an associated group of characteristic nuclear features, namely, marginated chromatin masses, spherical granular bodies of about $1\frac{1}{2}$ $\mu$ diameter and aggregations of dense particles. This close association indicates that the nuclear features are probably a peculiar manifestation of cytolysis in malignant cells of lymphoid origin, rather than the result of infection by an hypothetical virus as has sometimes been suggested. This conclusion is discussed.

### REFERENCES

BURKITT, D.—(1962*a*) *Ann. R. Coll. Surg. Engl.*, **30**, 211.—(1962*b*) *Post Grad. med. J.*, **38**, 71.—(1962*c*) *Nature, Lond.*, **194**, 232.

*Idem* AND O'CONOR, G. T.—(1961) *Cancer*, **14**, 258.

ITO, S.—(1962) Symposia of the International Society for Cell Biology. Vol. 1. " The Interpretation of Ultrastructure ". Edited by R. J. C. Harris. London and New York (Academic Press Inc.), p. 129.

LEPLUS, R., DEBRAY, J., PINET, J. AND BERNHARD, W.—(1961) *C.R. Soc. Biol., Paris*, **253**, 2788.

LUFT, J. H.—(1961) *J. biophys. biochem. Cytol.*, **9**, 409.

MILLONIG, G.—(1961) *J. appl. Phys.*, **32**, 1637.

O'CONOR, G. T.—(1961) *Cancer*, **14**, 270.

SHIPKEY, F. H. AND TANDLER, B.—(1962) In " Electron Microscopy ", Edited by S. S. Breese. New York (Academic Press Inc.), p. PP–9.

WATSON, M. L.—(1956) *J. biophys. biochem. Cytol.*, **2**, No. 4, suppl., 31.—(1958) *Ibid.*, **4**, 475.

---

### EXPLANATION OF PLATES

Both figures are electron micrographs of thin sections of biopsy material from Burkitt's lymphoma syndrome.

FIG. 1.—Survey picture showing parts of four degenerating cells grouped around an intercellular space (*s*) containing debris. Disruption of cell membranes is present (arrows) and there is swelling of the Golgi apparatus (*g*) and other cytoplasmic smooth membranous components, as at *x*. All four nuclei show electron-opaque masses of marginated chromatin and the nucleoplasm also contains aggregations of dense particles (*p*) and granular spherical bodies (*b*) with a dense centre. × 11,000.

FIG. 2.—Detail of a nucleus. A peripheral mass of chromatin surrounds an aggregation of dense particles (*p*) and a large spherical granular body (*b*) with characteristic dense central zone. × 37,000.

Epstein and Herdson.